# A meta-analysis on efficacy and safety of rituximab for neuromyelitis optica spectrum disorders

**DOI:** 10.1097/MD.0000000000030347

**Published:** 2022-09-09

**Authors:** Gu-Yi Dong, Yan-Hong Meng, Xiang-Jian Xiao

**Affiliations:** a Department of Neurology, Hebei General Hospital, Shijiazhuang, China; b Department of Neurology, Kailuan General Hospital, Tangshan, China.

**Keywords:** meta-analysis, neuromyelitis optica, neuromyelitis optica spectrum disorders, rituximab

## Abstract

To assess the efficacy and safety of rituximab (RTX) in the treatment of neuromyelitis optica spectrum diseases (NMOSDs), and give a guideline on clinical medication

**Methods:**

The databases of Pubmed, Embase, Cochrane Library, CNKI, and Wan fang were systematically searched by computer, and the search period was from the establishment of the databases until January 2022. To collect the trials of RTX in the treatment of NMOSDs, two researchers completed literature screening, quality assessment, and data extraction independently. Statistical analysis was performed using Review Manager 5.3 and Stata 15.1 software.

**Results:**

There were 37 studies in the meta-analysis, including 5 randomized controlled trials (RCTs) and 32 observational studies. Meta-analysis results revealed that NMOSDs patients treated with RTX significantly reduced the annualized relapse rate (ARR) (weighted mean difference [WMD] = 1.45, 95% confidence interval [CI]: 1.24–1.66, *P* < .01) and the Expanded disability status scale (EDSS) scores (WMD = 1.34, 95%CI: 1.25–1.44, *P* < .01). RTX is more effective than azathioprine (AZA) in the treatment of NMOSDs (ARR: WMD = −0.54, 95% CI: −0.75 to −0.33; EDSS: WMD = −0.65, 95% CI: −0.83 to −0.48; *P* < .0001).There was no difference in ARR and EDSS scores between anti-aquapor in-4-antibody seropositive NMOSD and seronegative NMOSD patients treated with RTX (ARR: WMD = −0.01, 95% CI: −0.25 to 0.24, *P* = .96 > 0.05; EDSS: WMD = 0, 95% CI: −0.30 to 0.31, *P* = .99 > 0.05). In this study, 681 patients were recorded safety data of RTX therapy, 23% (156 patients) had adverse events, and 0.7% (5 patients) of NMOSDs discontinued due to severe adverse reactions.

**Conclusions:**

NMOSDs patients treated with RTX can significantly reduce the relapse frequency and EDSS scores, and also improve neurological dysfunction, besides the efficacy is better than azathioprine. RTX has a high incidence of adverse reactions, which are mild and with certain self limited, it should be cautious in clinical medication

## 1. Introduction

Neuromyelitis optica spectrum disorders are a type of primary inflammatory demyelinating disease of the central nervous system. The patients are mainly female, and the onset is usually between 5 and 50 years old, with an average onset age of 39 years.^[1]^ At present, the pathogenesis of neuromyelitis optica spectrum diseases (NMOSD) is still unclear. Most scholars believe that aquaporin-4-antibody specifically binds to AQP-4 that causes necrosis of astrocytes, and releases of inflammatory mediators and infiltration of inflammatory response through complement dependent and antibody dependent cytotoxic pathways, which are eventually leading to injury of oligoendrocytes and demyelination.

Rituximab (RTX) is a human and mouse monoclonal chimeric antibody that binds to the CD20 antigen of B lymphocytes in the blood and initiates an immune response that mediates B cell lysis by depleting B lymphocytes. The study of Cabre et al^[2]^ and Kim et al^[3]^ showed that RTX could reduce the annual recurrence rate and the neurological dysfunction in patients with NMOSD. However, there are few randomized controlled trials (RCT) of RTX in the treatment of NMOSD, so it is necessary to conduct a meta-analysis on the efficacy and adverse drug reactions of RTX in the treatment of NMOSD, so as to provide evidence-based medicine basis for clinical medication.

## 2. Methods

### 2.1. Literature retrieval strategy

Embase, PubMed, The Cochrane Library, China National Knowledge Infrastructure (CNKI) database and Wan Fang Data were searched. The retrieval method is a combination of subject words (MesH table) and free words.

### 2.2. Inclusion and exclusion criteria

Inclusion criteria: The patients should meet the diagnostic criteria for neuromyelitis optic spectrum disease in 2015; RTX treatment; The language of the included literature was Chinese or English, and the study types included randomized controlled trial, cohort study, self-controlled before and after study, or case-control study; and Include at least one outcome indicator: Main indicators: Annual recurrence rate: the number of recurrences per year; Expanded Disability Status Scale score: EDSS is widely used in clinical evaluation of neurological dysfunction and disease severity in patients with NMOSD. The incidence of RTX adverse events (AEs) and discontinuation of treatment due to serious AEs. Secondary outcome indicators: Annual recurrence rate before and after treatment with RTX and AZA in patients with NMOSD; EDSS scores of NMOSD patients before and after treatment with RTX and AZA; The annual recurrence rate of patients with AQP-4 positive and AQP-4 negative NMO treated with RTX; EDSS scores of patients with AQP-4 positive and AQP-4 negative NMO treated with RTX.

Exclusion criteria: Case reports and review literature; ongoing studies, studies that did not provide outcome indicators, and duplicate studies; Exclude the literatures with the number of participants ≤ 5; unpublished literature; and Exclude the literatures whose annualized relapse rate (ARR) and EDSS data could not be extracted or whose full text could not be obtained.

### 2.3. Data extraction and quality evaluation

For continuous data (ARR and EDSS), crude data such as mean, standard deviation (SD), median, range, and sample size were directly extracted from the literature. The quality of the included RCT was assessed using the Cochrane Bias Risk Assessment Scale, and the literature quality of the non-RCT was assessed using the Newcastle-Ottawa Scale (NOS).

### 2.4. Statistical analysis

Review Manager 5.3 and Stata15.1 software were used for statistical analysis. Heterogeneity was detected by *Q* test and *I*^2^ test. *Q* test calculates chi-square value and *P* value, and the judgment standard is *P* value, *P* > .1, which indicates that the heterogeneity is small. If *I*^2^ < 50%, the fixed effects model was selected to combine the effect sizes of each study, and if *I*^2^ ≥ 50%, the random effects model was used. Sensitivity analysis, subgroup analysis or Meta regression were performed for outcome indicators with high heterogeneity to find out the reasons for the increase in heterogeneity. Since ARR and EDSS were continuous variable data, the Dersimonia–Laird (D–L) method^[4]^ was used to calculate weighted mean difference (WMD) and 95% CI. Z (*U*) test was used for hypothesis test, and the test level was set at .05. *P* < .05 indicated statistical significance. Otherwise, it was not considered statistically significant. Funnel plot method and EGGER were used to test whether there was any publication bias.

## 3. Results

### 3.1. Study identification and selection

**Figure 1.**
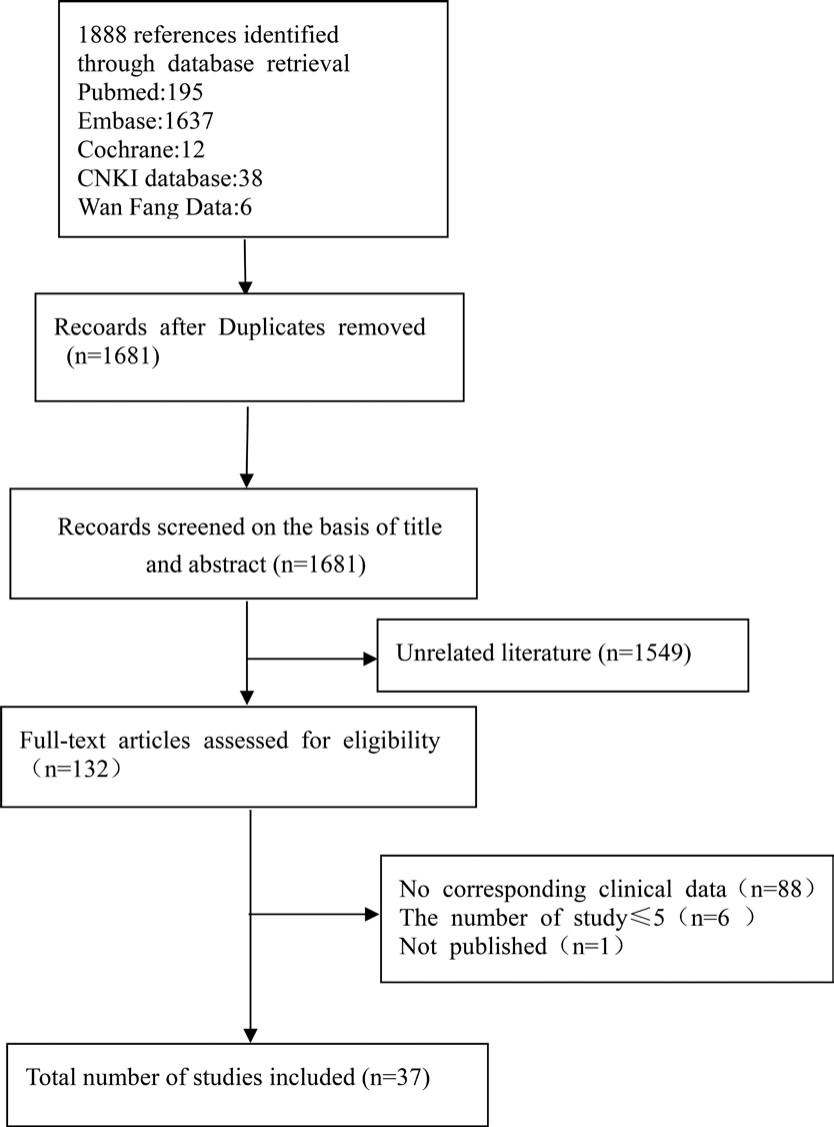
The selection process of documents.

By searching Chinese and English databases, a total of 1888 relevant literatures were retrieved. Finally, 37 studies were included in this systematic review. Figure 1 for the process of literature inclusion.

### 3.2. Basic characteristics of the literature included in this study

The data extracted in this study included the name of the investigator, the publication time of the literature, the number of subjects, gender, age, the number of AQP-4 antibody positive patients, the course of disease, and the follow-up time. Table 1 for the information of the included literatures.

**Table 1:** Main characteristics of the studies included in the meta-analysis.

Included studies	Research type	Patient no.	Sex (F/M)	Age (yr)	AQP4-Ab (+)	Duration of disease (yr/mo)	Follow-up (yr/mo)
Cabre et al 2018^[2]^	Prospective	32	30/2	39.9 (12.1)	20	NC	2 yr
Dastjerdi et al 2018^[5]^	Prospective	56	46/10	36.86	30	87.60 ± 59.65 mo	6/12 mo
Fernández-Megía et al 2015^[6]^	Retrospective	6	6/0	46 (38–58)	3	NC	NC
Jacob et al 2008^[7]^	Retrospective	25	22/3	38 (7–65)	14	4.5 (0.8–17) yr	19 (6–40) mo
Kim et al 2015^[3]^	Retrospective	100	92/8	43 (11)	94	11 (5) yr	67 (9–108) mo
Nikoo et al 2017^[8]^	RCT	33	29/4	35.33 (8.98)	13	6.23 (4.29) yr	12 mo
Shaygannejad et al 2019^[9]^	Prospective	44	35/9	37.2 ± 10.4	14	6.3 ± 4.1 yr	31.6 ± 7.3 mo
Zephir et al 2015^[10]^	Retrospective	32	27/5	45 ± 12.1	28	6.5 (1–410) mo	28.7 ± 21 mo
Zhang et al 2017^[11]^	Retrospective	31	23/8	42.2 ± 16.9	25	4.05 ± 2.11 yr	>2 yr
Annovazzi et al 2016^[12]^	Retrospective	73	64/9	46.5 ± 12.5	53	6 ± 7.2 yr	35.6 ± 27
Pellkofer et al 2011^[13]^	Prospective	9	8/1	36.1 (11.5)	9	11 (7.7) yr	29.6 (14.5) mo
Lindsey et al 2012^[14]^	Retrospective	8	7/1	37.6 (14.4)	4	65.1 (53.7) mo	39.9 (40.7) mo
Ip et al 2013^[15]^	Retrospective	7	6/1	52 (22–62)	4	57 (40–272) mo	24 (1–42) mo
Jeong et al 2016^[16]^	Retrospective	55	50/5	42 (15–68)	52	41.7 (2.1–231.5) mo	64.7 (6.2–99.8) mo
Collongues et al 2016^[17]^	Retrospective	21	19/2	37.8 (15.5)	19	46.9 (51.2) mo	31 (18) mo
Cohen et al 2017^[18]^	Prospective	40	33/7	40.2 (22–62)	20	40 (2–165) mo	2 yr
Yang et al 2018^[19]^	Prospective	20	19/1	40.7 (11.4)	10	11 (0.2–240) mo	29 (18–40) mo
Bedi et al 2011^[20]^	Retrospective	23	21/2	37.1 ± 14.6	15	114 (13–266) mo	32.5 (7–63) mo
Casallas-Vanegas et al 2020^[21]^	Retrospective	66	54/12	36.2 ± 12.01	44	5.85 ± 4.03 yr	NC
Correa Diaz et al 2019^[22]^	Retrospective	21	18/2	36.7 (13.3)	NC	NC	24.8 (6–47) mo
Flores et al 2012^[23]^	NC	13	12/1	33.3 ± 13.8	NC	NC	24 mo
Kim et al 2009^[24]^	NC	27	24/3	33.5 ± 11.6	NC	5.6 ± 3.6 yr	NC
Kim et al 2013^[25]^	Retrospective	30	27/3	38 (23–58)	NC	61 (49–82) mo	NC
Lin et al 2018^[26]^	RCT	14	NC	32.9 ± 13.6	NC	20.5 ± 6.8 mo	NC
Wu and Niu 2019^[27]^	Retrospective	15	13/2	31.8 ± 13.2	NC	1–144 mo	NC
Xiao et al 2020^[28]^	Retrospective	36	NC	NC	NC	NC	19.83 ± 7.74 mo
Bai 2016^[29]^	RCT	9	9	38.28	NC	NC	7–40 mo
Li 2019^[30]^	Retrospective	29	27/2	38.3 ± 13.9	27	NC	3.1/2.1 yr
Liu 2017^[31]^	RCT	15	15	37.84 ± 4.12	NC	6.93 ± 1.52 yr	NC
Niu 2019^[32]^	Retrospective	15	13/2	31.8 ± 13.2	NC	1–144 mo	NC
Wang et al 2018^[33]^	Prospective	21	20/1	26.2 ± 12.3	NC	9.2 ± 5.9 yr	28.4 ± 4.9 mo
Zhang 2016^[34]^	Prospective	19	NC	21-59	NC	NC	NC
Zhu 2018^[35]^	Retrospective	8	NC	48.52 ± 3.25	NC	NC	NC
Jia et al 2018^[36]^	RCT	9	8/1	42.78	NC	4.7 yr	NC
Zhang 2020^[37]^	Retrospective	29	25/4	29 (15–54)	24	39 (12–260) mo	NC
Cao et al 2021^[38]^	Retrospective	29	25/4	16 (55.2)	NC	NC	NC
Uzunköprü et al 2021^[39]^	Retrospective	85	69/16	43.78 ± 13.75	58	10.15 ± 7.93	21 mo

AQP4-Ab = aquaporin 4 antibody, NC = no clear, RCT = randomized clinical trial.

### 3.3. Quality evaluation

**Figure 2.**
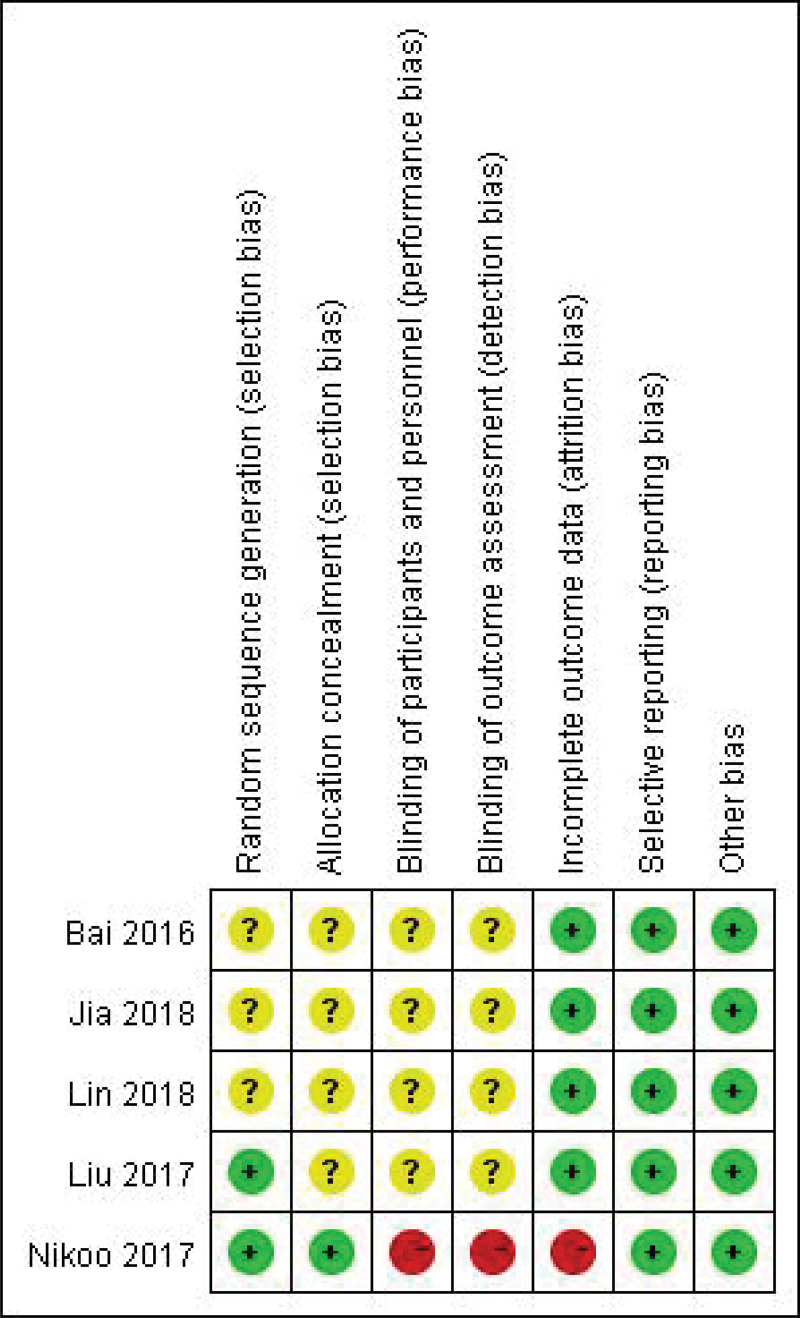
Risk of bias summary.

**Figure 3.**
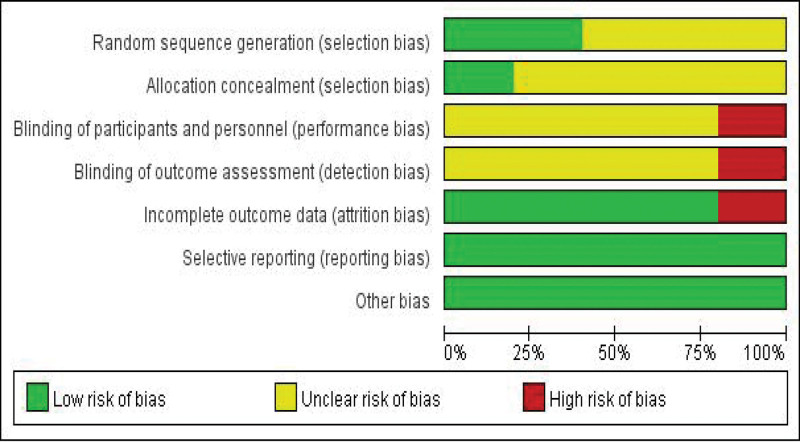
Risk of bias graph.

All RCTs included in this study met the evaluation criteria of medium literature quality, as shown in Figures 2 and 3. The Newcastle-Ottawa Quality Assessment Scale was used for the included case-control or cohort studies, as shown in Table 2.

**Table 2:** Evaluation of the Newcastle-Ottawa Scale for the assessment of the quality of nonrandomized studies.

Study	Selection	Comparability	Outcome
Prospective cohort study			
Cabre 2018	★★★	★★	★★★
Dastjerdi 2018	★★★	★★	★★
Shaygannejad 2019	★★★	★★	★★★
Pellkofer 2011	★★★	★★	★★★
Cohen 2017	★★★	★★	★★★
Yang 2018	★★★	★★	★★★
Wang 2018	★★★	★★	★★
Zhang 2016	★★★	★★	★★
Retrospective cohort study			
Fernández-Megía 2015	★★★	★★	★★
Jacob 2008	★★★	★★	★★
Kim 2015	★★★	★★	★★★
Zephir 2015	★★★	★★	★★★
Zhang 2017	★★★	★★	★★★
Annovazzi 2016	★★★	★★	★★★
Lindsey 2012	★★★	★★	★★★
Ip 2013	★★★	★★	★★★
Jeong 2016	★★★	★★	★★★
Collongues 2016	★★★	★★	★★★
Bedi 2011	★★★	★★	★★★
Casallas-Vanegas 2020	★★★	★★	★★
Correa Diaz 2019	★★★	★★	★★★
Kim 2013	★★★	★★	★★
Wu 2019	★★★	★★	★★
Xiao 2020	★★★	★★	★★
Li 2019	★★★	★★	★★★
Niu 2019	★★★	★★	★★
Zhu 2018	★★★	★★	★★
Zhang 2020	★★★	★★	★★
Cao 2021	★★★	★★	★★
Uzunköprü 2021	★★★	★★	★★

### 3.4. Results of meta-analysis

#### 3.4.1. Effects on ARR.

**Figure 4.**
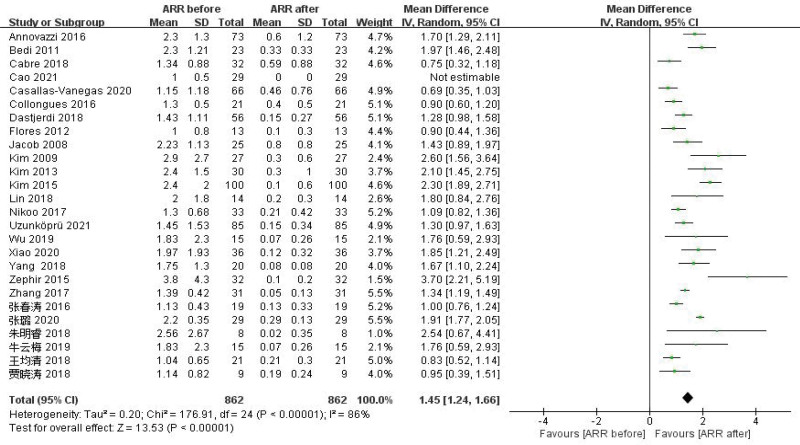
Forest plots of ARR. ARR = annualized relapse rate.

**Figure 5.**
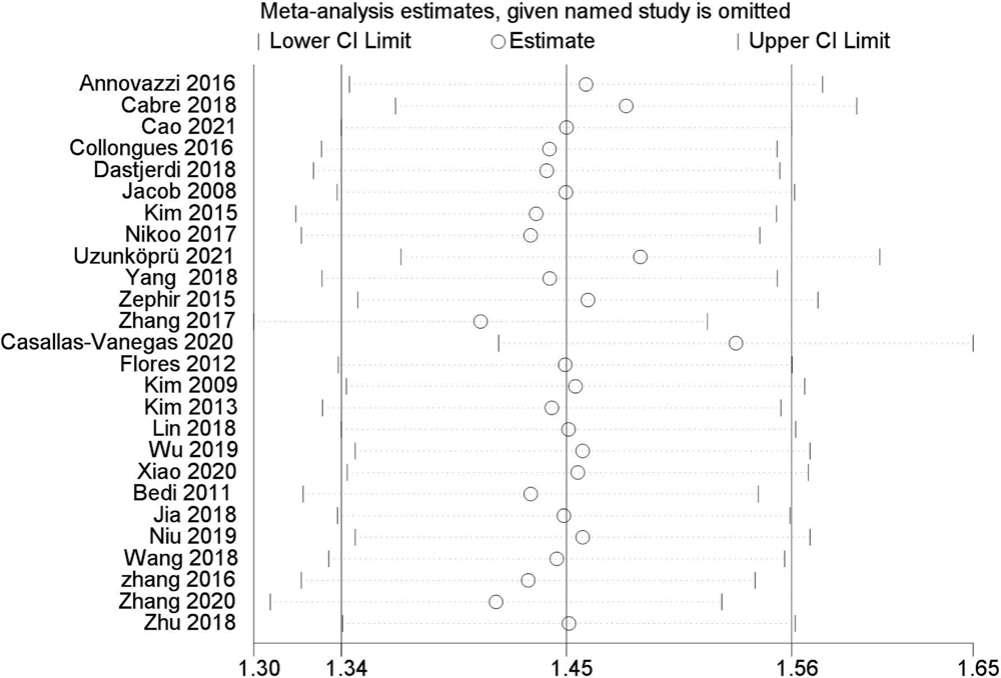
Sensitivity analysis of ARR. ARR = annualized relapse rate.

**Figure 6.**
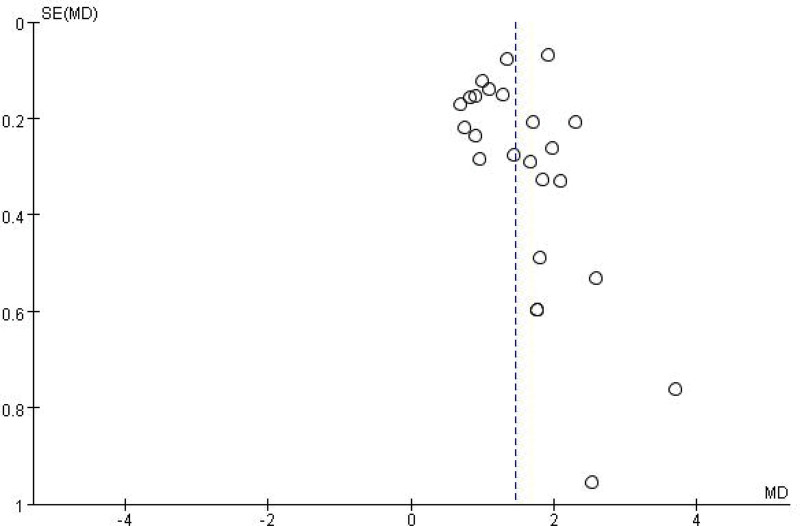
Bias figure of ARR. ARR = annualized relapse rate.

A total of 26 studies^[2,3,5,7,8,10–12,17,19–21,23–28,32–39]^ were reported ARR before and after RTX therapy in patients with NMOSD. The results of Meta-analysis showed that the ARR of NMOSD patients after RTX treatment was significantly decreased by 1.45, and the difference was statistically significant (WMD = 1.45, 95% CI: 1.24–1.66, *P* < .01). Figure 4 was for the forest plot. Since *I*^2^ = 86% > 50% and the *Q* test *P* < .1; it was suggested that there was a high degree of heterogeneity in the included literatures, and the random-effect model was selected for analysis. In order to ensure the stability of the study, sensitivity analysis was carried out, and the results showed that there was no significant interference from any literature on the results of this meta-analysis, indicating that this study had good stability. Figure 5 was for sensitivity analysis. Next, Meta regression was continued to investigate the factors influencing effect size, in that effect size was the dependent variable and age and EDSS baseline were independent variables. No significant correlation was found between the ARR difference and the following variables: age (*P* = .32; 95% CI: −0.02 to 0.07), baseline EDSS (*P* = .31; 95% CI: −0.098 to 0.31). Funnel plots were drawn by RevMan 5.3 software to investigate the existence of publication bias, as shown in Figure 6. It can be clearly seen from the funnel plot, which is asymmetric. Egger test was conducted with Stata 15.1 software, and the result showed that *P* = .004 < 0.1, so it was judged that there was publication bias in the literature of this study.

#### 3.4.2. Efficacy on the EDSS score.

**Figure 7.**
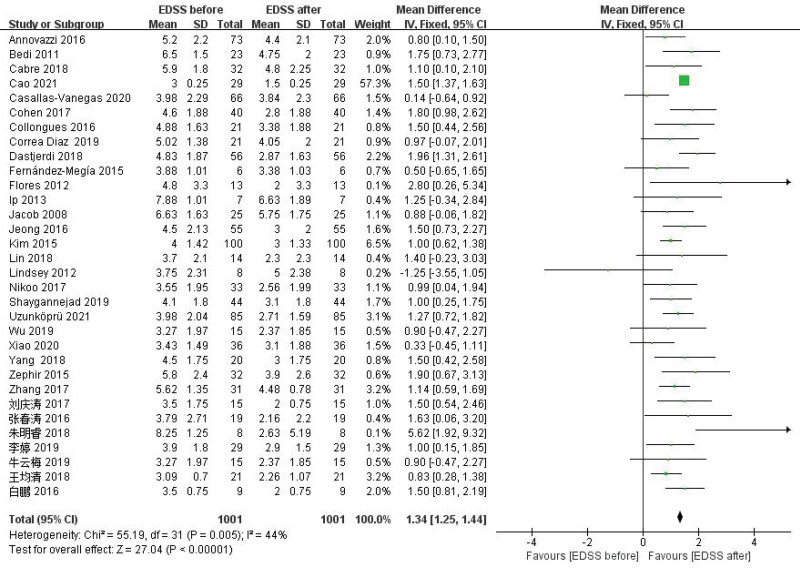
Forest plot of EDSS. EDSS = expanded disability status scale.

**Figure 8.**
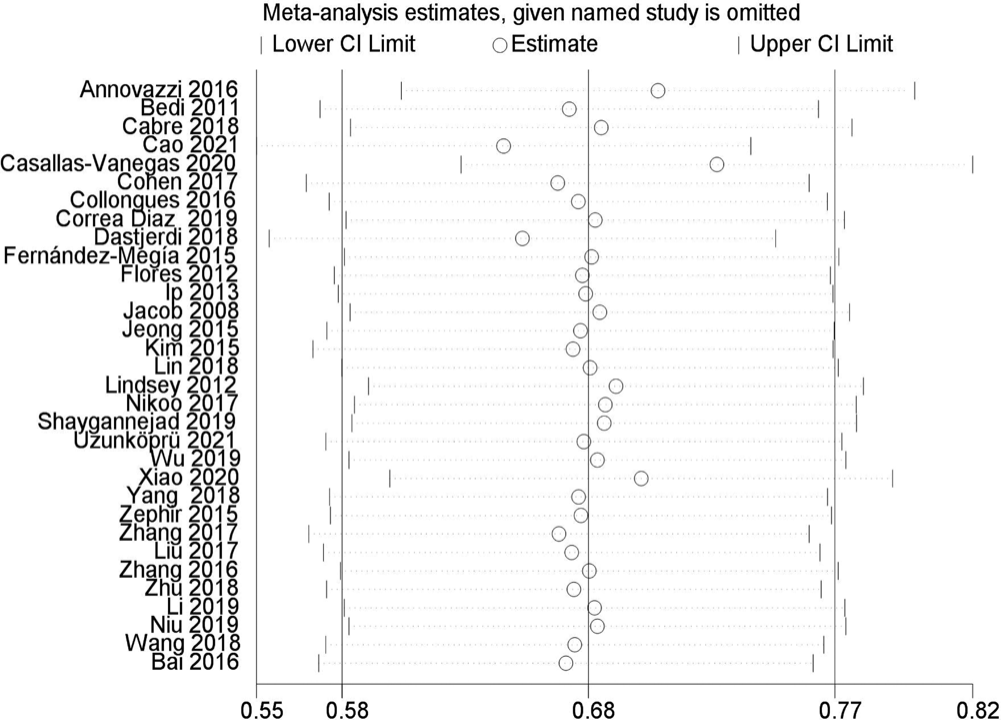
Sensitivity analysis of EDSS. EDSS = expanded disability status scale.

**Figure 9.**
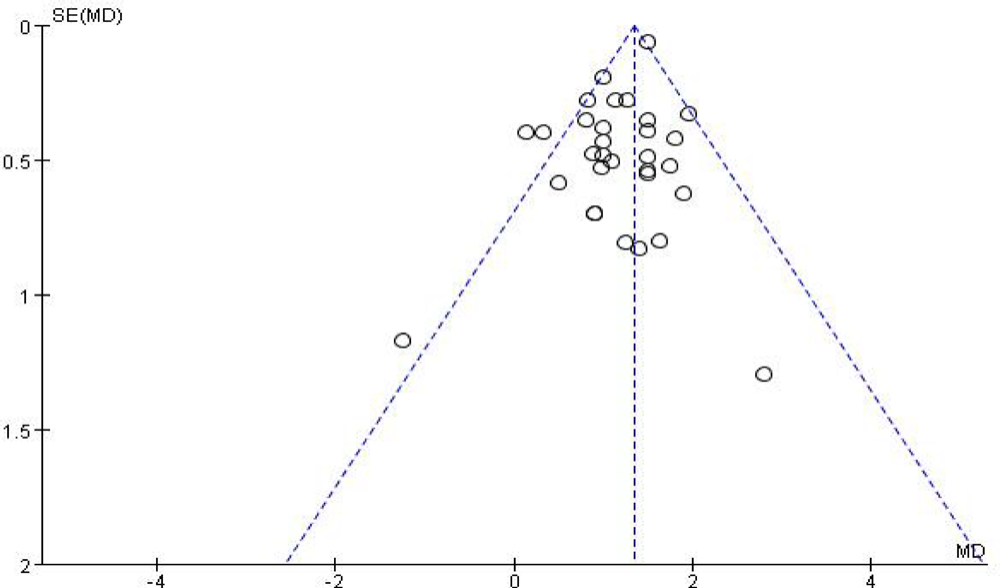
Bias figure of EDSS. EDSS = expanded disability status scale.

Meta-analysis results showed that the average EDSS score of NMOSD patients after RTX treatment decreased by 1.34 points, and the difference was statistically significant (WMD = 1.34, 95% CI: 1.25–1.44, *P* < .01). Figure 7 for the forest plot. Since *I*^2^ = 44% < 50%, it was suggested that there was a low degree of heterogeneity in the included literatures, and the fixed-effect model was selected for analysis. The results of sensitivity analysis showed that there was no significant interference from any literature on the results of this meta-analysis, indicating that the results of this meta-study were stable. Figure 8 for Sensitivity analysis of EDSS. The funnel plot of this study is shown in Figure 9. It could be clearly seen from the funnel plot that it is asymmetric. At the same time, Egger test was conducted with Stata 15.1 software, and the result showed that *P* = .039 < .1, so it was judged that there was publication bias in the literature of this study.

### 3.5. Comparison of efficacy between RTX and AZA in the treatment of NMOSD

**Figure 10.**
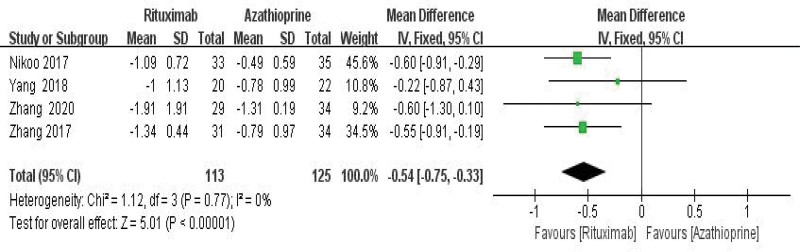
Forest plot of ARR comparison between rituximab and azathioprine. ARR = annualized relapse rate.

**Figure 11.**
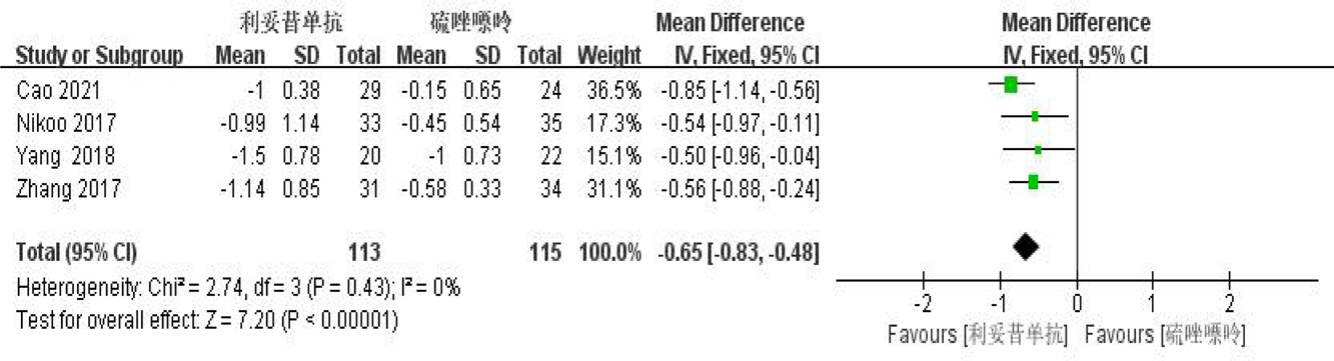
Forest plot of EDSS comparison between rituximab and azathioprine. EDSS = expanded disability status scale.

RTX was better than azathiopine in the treatment of NMOSD and significantly reduced ARR (WMD = −0.54, 95% CI: −0.75 to −0.33, *Z* = 5.01, *P* < .01) and EDSS score (WMD = −0.65, 95% CI: −0.83 to −0.48), with *Z* = 7.20, *P* = .0001 < .01). Its forest plot could be seen in Figures 10 and 11. Since *I*^2^ = 0% < 50%, it was suggested that there was no heterogeneity in the included literatures. Egger test was conducted with Stata 15.1 software, and the results showed that (ARR *P* = .526 > 0.1, EDSS *P* = .423 > .1), so it was judged that there was no publication bias.

### 3.6. Efficacy of RTX in patients with anti-aquapor in-4-antibody-seropositive NMO and seronegative NMO

**Figure 12.**
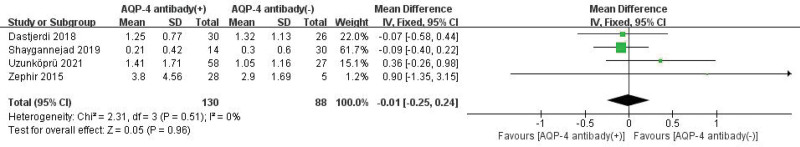
Forest plot of ARR comparison between patients with AQP-4 positive and negative NMO treated with rituximab. ARR = annualized relapse rate, AQP-4 = anti-aquapor in-4-antibody, NMO = neuromyelitis optica.

**Figure 13.**
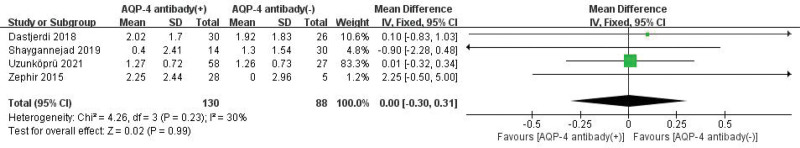
Forest plot of EDSS comparison between patients with AQP-4 positive and negative NMO treated with rituximab. AQP-4 = anti-aquapor in-4-antibody, EDSS = expanded disability status scale, NMO = neuromyelitis optica.

ARR comparison results showed that there was no statistical significance between patients with anti-aquapor in-4-antibody-seropositive NMO and seronegative NMO (WMD = −0.01, 95% CI: −0.25 to 0.24, *Z* = 0.05, *P* = .96 > .05). EDSS comparison results showed that there was no statistical significance between patients with anti-aquapor in-4-antibody-seropositive NMO and seronegative NMO (WMD = 0, 95% CI: −0.30 to 0.31, and *Z* = 0.02, *P* = .99 > .05). Figures 12 and 13 were for the forest plot. Since *I*^2^ = 0% < 50% and *I*^2^ = 30% < 50%, it was suggested that there was a low degree of heterogeneity in the included literatures. Egger test was conducted with Stata 15.1 software, and the results showed that ARR: *P* = .850 > .1, EDSS *P* = .535 > .1, suggesting that there was no publication bias among the studies.

### 3.7. Safety

A total of 1105 people were enrolled in 37 studies, of which 681 patients documented safety data for RTX therapy. AEs occurred in 156 patients (23%), including 5 patients (0.7%) who were discontinued due to severe adverse reactions. The most common adverse reactions were infusion reactions and infection. Table 3 was for specific information.

**Table 3:** Adverse events on RTX in neuromyelitis optica spectrum disorders.

Study	Total number of patients	Number of patients with adverse events	Adverse events	Number of events	Total number of discontinuation due to adverse effects
Cabre 2018	32	7	Lower urinary tract infection	1	1
			Pharyngitis	1	
			Xerostomia	1	
			Moderate headache	1	
			Moderate fever	1	
			Laryngeal dysesthesia	1	
Fernández-Megía 2015	6	2	Infusion reactions	2	–
Jacob 2008	25	12	Infusion reactions	7	–
			Herpes simplex and positive tuberculin skin test herpes zoster	1	
			Recurrent Clostridium difficile colitis	1	
			A cutaneous fungal infection	1	
			Fatal urinary tract–related septicemia	1	
				1	
Kim 2015	100	34	Infusion-related reactions	26	–
			Herpes zoster infection	5	
			Pneumonia	2	
			Thyroid cancer	1	
Nikoo 2017	33	4	Allergic reactions	4	1
Shaygannejad 2019	44	15	Infusion reactions	14	1
			Severe allergic reaction	1	
Zephir 2015	32	0	No relevant side effect	0	–
Zhang 2017	31	4	Infusion reactions	4	–
Annovazzi 2016	73	19	Urinary tract infections	6	2
			Respiratory infections	4	
			Infusion reactions	7	
			Died	2	
Pellkofer 2011	9	4	Urogenital infection, thrombosis, death	1	–
			Adnexitis	1	
			Pneumonia	1	
			Urosepsis	1	
Ip 2013	7	2	Infusion reactions	2	–
Yang 2018	20	1	Transit hyperpyrexia	1	–
Bedi 2011	23	7	Herpes zoster infection	1	–
			Urinary tract infection	1	
			Respiratory infections	2	
			Fatigue	1	
			Transient leukopenia	1	
			Transient elevations of hepatic transaminases	1	
Casallas-Vanegas 2020	66	3	Respiratory infections	2	–
			Urinary tract infection	1	
Flores 2012	13	3	Tetter	3	–
Kim 2013	30	12	Infusion reactions	12	–
Lin 2018	14	2	Phthisis	1	1
			Respiratory infections	1	
Wu 2019	15	3	No clear		–
Xiao 2020	36	8	Infusion reactions	5	–
			Urinary tract infection	3	
Jia 2018	9	2	Infusion reactions	1	–
			Herpes zoster infection	1	
Niu 2019	15	1	Respiratory infections	1	–
Wang 2018	21	8	No clear		–
Zhang 2016	19	1	Herpes zoster infection	1	–
Zhu 2018	8	2	Infusion reactions	2	–

## 4. Discussion

NMOSD is an inflammatory autoimmune demyelinating disease of the central nervous system, and tends to occur in young people, which with other autoimmune diseases, such as systemic lupus erythematosus, sjogren syndrome, myasthenia gravis, thyroid function hyperfunction, hashimoto thyroiditis, mixed connective tissue disease, tuberous polyarteritis, and so on. Antinuclear antibodies, anti-SSA/SSB antibodies, and anti-cardiolipin antibodies could be detected in serum. Although relatively unknown due to the small number of patients with NMOSD, significant progress has been made in recent years in the study of NMOSD, such as the discovery of MOG-Ab, the NMOSDs diagnostic criteria developed by the International NMO Diagnostic Group (IPND) in 2015, and the development of new drugs, which deepened people’s understanding of NMOSDs. It is worth noting that NMOSD has a risk of recurrence and accumulation of disability. If NMOSD is not treated, nearly half of the patients will develop limb weakness and blindness, and one-third of the patients may die within 5 years.^[40]^ Therefore, prevention of recurrence has become the focus of treatment. Moreover, many studies have shown that certain immunosuppressants can reduce the recurrence of NMOSD and the degree of disability.

At present, the clinical treatment of NMOSD includes acute treatment, sequential treatment (immunosuppressive therapy), symptomatic treatment, and rehabilitation treatment, which can effectively improve the condition of patients. If NMOSD patients have symptoms of neurological impairment or relapse, not only acute treatment is needed, but also the main treatment methods should include glucocorticoid, plasma exchange, intravenous immunoglobulin, etc, and are needed to reduce clinical symptoms and prevent and treat complications. In contrast to Disease modification therapy for multiple sclerosis, sequential treatment of NMOSD is required to prevent recurrence, so patients with AQP-4 antibody positive NMOSD or those with AQP-4 antibody negative NMOSD should be treated with immunosuppressant therapy, which actively reduces the annual recurrence rate, the degree of disability accumulation, and improves the prognosis. Due to the lack of effective means to distinguish between single-phase and multi-phase NMOSD at present, excessive immune intervention is not necessary for AQP4-IgG negative single-phase NMOSD. At present, Mycophenolate mofetil (MMF), AZA, RTX, and Methotrexate (MTX) are mainly used clinically, which can effectively prevent NMOSD recurrence, but these have not been verified by high quality RCT. Espiritu and Pasco^[41]^ have published an article, which showed that AZA improves relapses and disability in patients with NMOSD. Damato et al^[42]^ and Mirmosayyeb et al^[43]^ also have published an article titled A systematic review and meta-analysis of Efficacy and Safety of Rituximab Therapy in Neuromyelitis Optica Spectrum Disorders, which showed that RTX therapy reduces the frequency of NMOSD relapses and neurological disability in patients with NMOSDs.

RTX is the first anti-CD20 monoclonal antibody approved for the treatment of B-cell lymphoma in 1997. B cells are usually almost completely depleted two weeks after the infusion of RTX, which lasts 6 to 12 months. Therefore, patients with NMOSD should receive regular maintenance therapy every 6 months. However, there were significant differences in the initial dose of RTX required for B cell depletion and the time to B cell reproliferation in patients. In a study of patients with NMOSD, it took 17% of patients 6 months to regain B cells.^[44]^However, B-cell consumption lasting more than 3 years after the administration of RTX has also been reported,^[45]^ so the administration time should be set according to the situation of B-cell reproliferation to prevent overtreatment of patients with persistent B-cell failure, so as to prevent complications and reduce costs. The results of this meta-analysis proved that RTX could significantly reduce the ARR and EDSS scores of NMOSD patients, and its therapeutic effect was better than AZA. There was no statistical difference between RTX in the treatment of NMOSD patients with anti-aquapor in-4-antibody (AQP-4)-seropositive and seronegative. In addition, the results show that 156 cases (23%) experienced AEs, and 5 cases (0.7%) with severe adverse reactions caused by drug withdrawal, which indicated higher incidence of adverse reactions in RTX, but the vast majority of AEs for mild-to-moderate, have certain self-limited. It should be careful to use in the clinical application of rituxan for NMOSD patients.

This meta-analysis evaluated the efficacy and safety of RTX in the treatment of NMOSD from 37 included studies. In addition, the study assessed the efficacy of RTX and AZA in the treatment of NMOSD, indicating that there were no clinical differences between patients with AQP-4 positive and AQP-4 negative neuromyelitis optic spectrum disease treated with RTX. We could get some updated conclusion and help to develop a more reasonable treatment in clinical work.

### 4.1. Limitations

Although the quality of all the included studies was moderate, there were differences in terms of intervention and protocol design, and the results may have some limitations. There is publication bias in the influence of RTX on the recurrence rate and EDSS of NMOSD patients, which may be related to the high heterogeneity among included studies and the small sample size of some studies. Methods such as inclusion of high-quality homogeneous studies and expansion of sample size can reduce publication bias and make the results more accurate. Besides, there are few RCT on the efficacy and safety of RTX in the treatment of NMOSD, so more large multicenter RCTs are needed to further verify the accuracy of the conclusions.

## Acknowledgments

The authors would like to thank Professor Wang for his help in designing the search strategies.

## Author contributions

G-YD and X-JX designed the analysis. G-YD and X-JX collected and abstracted the data and carried out the statistical analysis. G-YD drafted the manuscript. All authors analyzed and interpreted the data and critically revised the manuscript for important intellectual content. The contents of this study are solely the responsibility of the authors and do not necessarily represent the official view of their institutions or any other party. G-YD and X-JX have full access to all the data and take full responsibility for the data, the analysis and the interpretation. All authors reviewed and approved the final report.

Conceptualization: Xiang-Jian Xiao.

Data curation: Gu-Yi Dong.

Formal analysis: Gu-Yi Dong.

Investigation: Gu-Yi Dong.

Methodology: Gu-Yi Dong.

Software: Xiang-Jian Xiao.

Writing – original draft: Gu-Yi Dong, Yan-Hong Meng, Xiang-Jian Xiao.

Writing – review & editing: Gu-Yi Dong, Yan-Hong Meng.
